# Decline in diversity of tropical soil fauna under experimental warming

**DOI:** 10.1098/rspb.2024.2193

**Published:** 2024-12-11

**Authors:** Hubert A. Szczygieł, Orpheus M. Butler, Andrew T. Nottingham

**Affiliations:** ^1^ Smithsonian Tropical Research Institute, Panamá Apartado Postal 0843-03092, República de Panamá; ^2^ Australian Rivers Institute, Griffith University, Nathan, Queensland 4111, Australia; ^3^ School of Geography, University of Leeds, Leeds, UK

**Keywords:** tropical, soil, biodiversity, climate change, warming, insect decline

## Abstract

Climate change is exacerbating a global decline in biodiversity. Numerous observational studies link rising temperatures to declining biological abundance, richness and diversity in terrestrial ecosystems, yet few studies have considered the highly diverse and functionally significant communities of tropical forest soil and leaf litter fauna. Here, we report major declines in the order-level richness and diversity of soil and leaf litter fauna following three years of experimental whole-profile soil warming in a tropical forest. These declines were greatest during the dry season, suggesting that warming effects could be exacerbated by drought. Contrary to findings from higher latitudes, total faunal abundance increased under warming, and these effects were paralleled by major shifts in community composition. These responses were driven by increased dominance of a relatively small number of thermophilic taxa, and of oribatid mites in particular. Our study provides direct experimental evidence that warming causes diversity declines and compositional shifts for tropical forest soil and leaf litter fauna, a result with potential consequences for soil functions and biogeochemical cycles, and that highlights the vulnerability of tropical biodiversity to climate change.

## 1. Introduction

Mounting evidence indicates a global decline in the diversity and abundance of terrestrial arthropods over recent decades [1–9]. Numerous studies point to climate warming as a leading cause of these declines [1,8–11], yet such studies have mostly been carried out in high-latitude biomes such as temperate deciduous forests and arctic tundra. By comparison, low-latitude biomes such as tropical forests—which contain exceptionally high levels of arthropod diversity [12]—have received little attention. Temperatures in the tropics are predicted to exceed their historically stable levels faster than those at higher latitudes [13], and tropical arthropods are expected to be particularly sensitive to warming owing to their narrow thermal tolerances [8,11,14,15], which are likely a result of the historically low variability of tropical climates [11]. Although there are a small number of reported declines in the diversity and abundance of tropical arthropods [5,6,16], and some reports of no decline [17,18], to our knowledge all such studies were based on observational rather than experimental data and were mostly restricted to Lepidoptera and their parasites. Thus, the responses of tropical arthropod communities to a warming world, and the associated consequences for ecosystem functioning, remain poorly understood.

The ability of arthropods to adapt to climate change is related to their thermal and desiccation tolerances. Arthropods have developed many strategies for thermal buffering within their thermal tolerance limits, but the plasticity of those limits themselves is limited and evolutionary capacity to increase limits differs widely among taxa [19]. While warming could be beneficial to some groups [17,20], it is expected that climate change will push many arthropods beyond thermal maxima [11,21]. Research on tropical butterflies indicates that these thermal maxima are inversely related to thermal buffering ability across species [22], suggesting that climatic warming poses a greater risk to arthropod species that maintain steady body temperature than to species that can fluctuate their body temperature to buffer the effect of ambient temperature change. This aligns with the climatic variability hypothesis [23]: species that experience greater temperature variability have a greater range of thermal tolerance. One would expect soil arthropods to have especially narrow thermal tolerance owing to the high thermal stability of soil. However, the consequences of climate warming for tropical arthropods are unclear for the communities of arthropods inhabiting soil and leaf litter environments [24,25]. These animals are critical to ecosystem function, playing both direct and indirect roles in decomposition [26], carbon (C) and nutrient cycling [27–29] and primary production [30]. Moreover, the activities of certain functionally significant taxa, such as termites, can buffer tropical forests against the ecological and biogeochemical consequences of the protracted drought events that increasingly threaten tropical forests as the global climate changes [31,32]. Thus, the response of tropical soil and leaf litter arthropods to warming could have wide-reaching consequences for overall ecosystem structure and function that feed back to the global climate system.

There is currently a lack of direct experimental evidence of tropical arthropod community responses to climate change [33]. One study in Puerto Rico used canopy trimming and biomass addition to experimentally test responses of fauna to changes in microclimate [34]. These microclimate changes included changes in throughfall, soil and litter moisture, temperature, light and substrate availability, which resulted in a decrease in the diversity of soil fauna [34]. However, the vast majority of studies on terrestrial arthropod responses to climate change have been based on observational data across environmental gradients [14,32] and long-term monitoring data [1,3–5], with potential for confoundment by covarying changes in other environmental variables, decadal-scale community fluctuations and a lack of reliable baseline information [35]. Experimental soil warming studies of arthropod communities have been restricted to high-latitude and cool alpine regions [36–46]. Two of these studies reported a decline in soil arthropod taxonomic richness with warming [42,43], but in most cases, the biodiversity of soil and/or leaf litter entire arthropod communities was either unaffected by warming [36,38–40,44,47,48] or not given explicit consideration [37,41]. For example, in sub-Arctic grassland soils that were geothermally warmed, the hexapod community composition changed after 6 years [45], while Collembola functional diversity declined after six years but returned to control levels after 50 years [46]. In contrast to the weak correlation of experimental soil warming with soil fauna diversity, more consistent responses have been shown for soil arthropod abundance, biomass and activity. Arthropod abundance and biomass have been generally shown to decrease in response to experimental warming in forests [41,42], grasslands [39,45] and peatlands [43]. Despite these observed decreases in abundance and biomass, warming has also been shown to increase soil faunal activity, including fungal grazing [39,49,50]. Thus, higher activity rates may mediate the effects of decreased abundance and biomass on ecological function.

Here, we tested the hypothesis that warming of a lowland tropical forest will cause a decline in the order-level richness, diversity and abundance of soil and leaf litter fauna. Given the historically stable tropical climate [11,14] and notionally narrow thermal tolerances of tropical arthropods [11,14,21], we predicted that the responses in our tropical site would differ from those observed at higher latitudes [39,41,42]. We further predicted shifts in the overall composition of soil and leaf litter communities owing to differences among taxa in their vulnerability to warming. Indeed, a preliminary study, carried out after 2 years of warming, identified a warming-induced shift in the composition of ant communities at our study site [51], and this could reflect wider changes in the composition of soil and leaf litter faunal communities. We use the term ‘fauna’ to encompass all animals collected in this study, comprising the meso- and macro-fauna of soil and leaf litter, which together form a major part of terrestrial detrital food webs. This group is largely composed of arthropods, although gastropod molluscs and annelids are present at low abundances.

The study was performed on an *in situ* whole-profile soil warming experiment situated on Barro Colorado Island (BCI), Panama, where belowground heating cables were used to heat the entire soil profile by 4°C (electronic supplementary material, figure S1; [52]). The experiment is composed of ten 20 m^2^ plots in a paired design for a total of five warmed–control replicates. After 3 years of continuous soil warming, and over a full seasonal cycle, we collected *ca* 72 000 organisms residing in soil and leaf litter habitats and identified them to Order (see §4 for further detail on experimental design, collection and identification protocols).

## 2. Results

**Figure 1.**
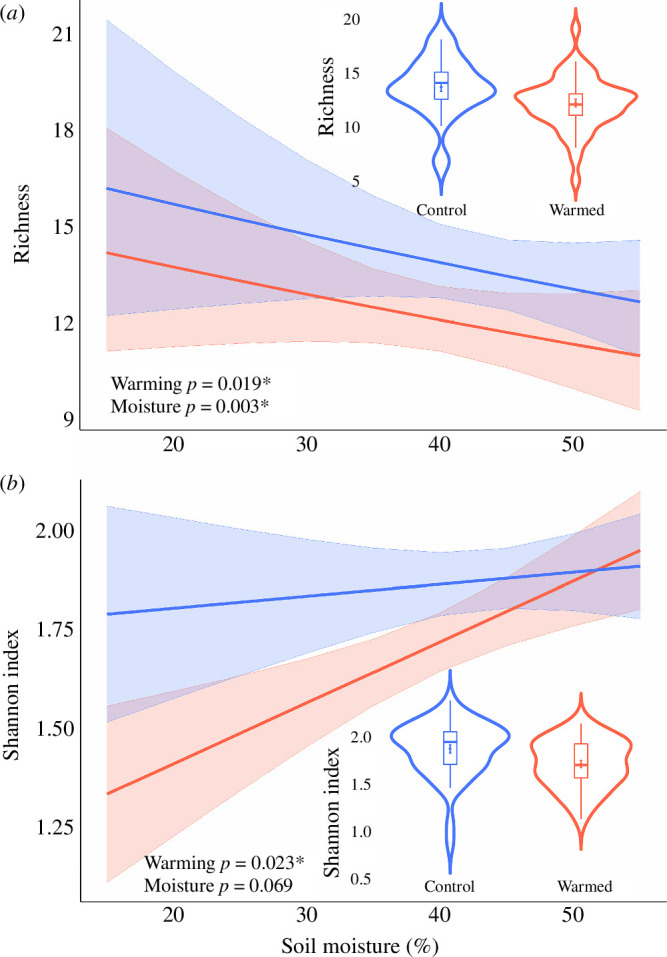
The order-level diversity and richness of soil fauna following 3 years of experimental soil warming. (*a*) Mean taxonomic richness. (*b*) Mean Shannon diversity. Data are for the entire sampling period (14 December 2019–1 October 2020). Box plots are standard Tukey plots, where the centre line represents the median, the lower and upper hinges represent the first and third quartiles and whiskers represent +1.5 the interquartile range, and the point represents the mean with standard error bars. Blue lines represent control plots, while red lines represent warmed plots. The difference in means between warmed and control plots for (*a*) is 8.5% and for (*b*) is 10.9%. Linear models show significant (*p* < 0.05) effects of warming and moisture on diversity; however, warming alone is a significant predictor of richness. The *p* values are given for warming and moisture effects with significance denoted by asterisks: *p* < 0.05 (*), *p* < 0.01 (**).

**Figure 2.**
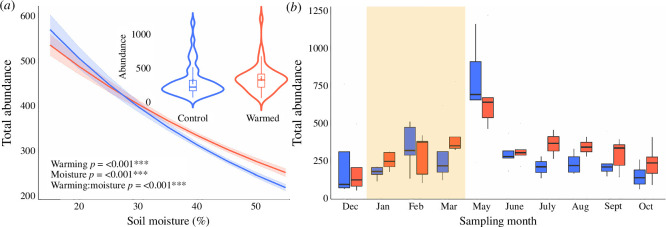
The effect of 3 years of experimental soil warming on soil fauna abundance. (*a*) Mean total faunal abundance over the entire sampling period, from December 2019 to October 2020. (*b*) Mean total faunal abundance by month. The total abundance of fauna in warmed plots was 15.8% greater than that in control plots. Total faunal abundance was 21.4% higher than controls during the dry season and 13.4% lower during the wet season. Box plots are standard Tukey plots, where the centre line represents the median, the lower and upper hinges represent the first and third quartiles, and whiskers represent +1.5 the interquartile range. Blue lines and box plots represent control plots, while red lines and box plots represent warmed plots. The shaded area in (*b*) represents the dry season (1 January to 1 April) where precipitation was < 50 mm per month. Warming treatment, soil moisture and the treatment × moisture interaction were all significant predictors of total invertebrate abundance in linear mixed effects models. Total abundance increased during the dry season, with a peak at the beginning of the wet season and a subsequent drop throughout the wet season, while total abundance was consistently greater in warmed plots. Total abundance decreased with moisture in both warmed and control plots across all sampling dates, as seen in (*a*), with abundance in control plots greater than that in warmed plots when moisture was low. The *p* values are given for warming and moisture effects in (*a*), with significance denoted by asterisks: *p* < 0.001 (***).

**Figure 3.**
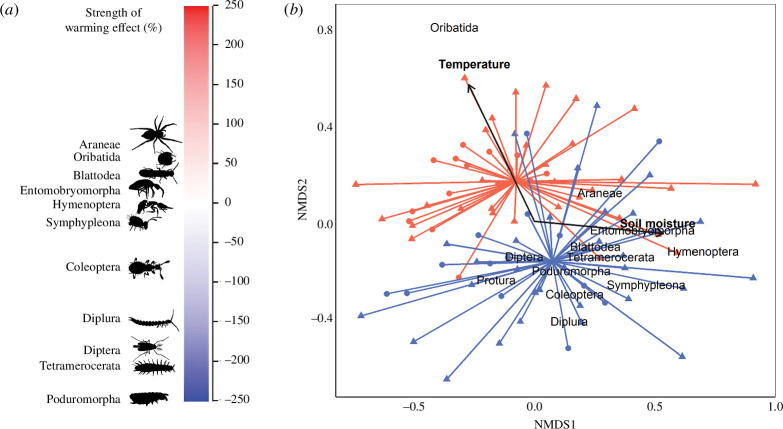
Soil faunal taxon-specific responses and changes in community composition after 3 years of experimental soil warming. Results are based on non-metric multidimensional scaling (NMDS) ordination of soil invertebrate communities based on Bray–Curtis dissimilarities. (*a*) The strength of the warming effect (250% to −250%) on the response of specific taxa, where positive values indicate taxa were more abundant on warmed plots, and *vice versa*. (*b*) NMDS spider diagram; *k* = 2; stress ≈ 0.2. Each point on the spider diagram represents the order-level community composition for one plot on one sampling date, with warmed plots coloured red and control plots coloured blue, circles representing the dry season and triangles representing the wet season. Relationships between taxa and treatments are represented by distance between taxon names. Significant predictors are overlaid as vectors. The direction of each vector indicates the direction of the gradient, while length indicates the strength of the correlation. Community composition was significantly different between treatments (*p* < 0.001; Permutational analysis of variance).

Three years of *in situ* soil warming led to major changes in the communities of both soil and leaf litter fauna (figures 1–3). The richness of taxonomic orders (i.e. the number of distinct orders) of soil fauna was reduced by 10.9% in warmed plots (*p* < 0.05; figure 1*a*), while the Shannon diversity (which represents both taxonomic richness and ‘evenness’) of soil fauna was reduced by 8.5% (*p* < 0.05; figure 1*b*). A similar response was evident for leaf litter fauna, for which taxonomic richness and Shannon diversity, respectively, declined by 5.3% and 12.1% under warming. The total abundance of both soil fauna and leaf litter fauna increased in the warmed plots (effect sizes of 15.8% for soil and 14.6% for litter; *p* < 0.001; figure 2). However, the abundance responses of specific taxa varied widely (electronic supplementary material, figure S4). For example, the abundances of Oribatida, Symphyla and Blattodea (the latter comprised almost entirely of termites) increased under warming (effect sizes ranging from 34%−70% for soil and from 35%−83% for leaf litter; treatment *p* < 0.001; electronic supplementary material, tables 1 and 2), while the abundances of Coleoptera, Psocoptera, Protura, Diplura, Pseudoscorpiones, Diplopoda, Chilopoda, Isopoda, Tetramerocerata and several other taxa declined (effect sizes ranging from 33%−1851% for soil and from 34%−1015% for leaf litter; treatment *p* < 0.001; electronic supplementary material, tables S1 and S2). This diverse suite of taxon-specific abundance responses to warming translated to a major shift in the overall composition of soil and leaf litter communities (*p* < 0.05; figure 3; electronic supplementary material, figure S3), characterized by an increased dominance of a small subset of taxa (notably oribatid mites) alongside declines in numerous other taxa. To confirm that our findings were not skewed by rare taxa, we reran models for abundance, richness and diversity excluding 14 orders that only occurred in <10% of soil samples, and found that trends remained significant (*p* < 0.05).

## 3. Discussion

Our results provide clear evidence that the biodiversity of tropical soil and leaf litter fauna can be negatively affected by increased soil temperatures and support the view that warming has contributed to the declines in arthropod diversity observed in recent decades [3–6,9]. At the same time, the striking contrast between our findings and those of temperate warming experiments, in which richness and diversity have rarely been affected [36–42,44,46–48] and abundances have often declined [39,41,42], are consistent with predictions that arthropod diversity may be more sensitive to a warming climate in lower latitudes [11,14]. Warming effects on species-level biodiversity may be larger than those we observed at order level, particularly in the case of the Shannon index, because large increases in mite abundance would correspond to declines in species evenness across the entire arthropod assemblage. Indeed, several of the taxa that were negatively affected by warming in our study represent some of the most species-rich orders of animals of Earth (e.g. Coleoptera and Hymenoptera). By extrapolating from the species : abundance ratios for arthropod orders in a nearby tropical forest site (San Lorenzo Protected Area, *ca* 20 km NW of BCI), we estimate that the loss of 1−2 evidently vulnerable orders under warming (e.g. Coleoptera, Diptera and Poduromorpha), the potential for which is highlighted in figure 3*a*, could correspond to a 0.4%−13.4% decline in total species richness for soil and a 0.3%−29.4% decline in total species richness for litter, depending on which orders are lost. Although preliminary and determined indirectly, this estimate for large but variable species declines under warming highlights the need for further direct assessments of species-level diversity responses to warming in the tropics.

The responses of taxonomic richness and diversity to warming observed in our study provide a new perspective on the prominent hypothesis that tropical biodiversity declines under warming are driven by the narrow thermal tolerance of tropical organisms [11,14]. While the losses of ordinal richness likely reflected local absences of various rare taxa, the declines of Shannon diversity were wing to losses of richness in combination with losses of ‘evenness’ via disparate changes in relative abundances of prevalent taxa (figure 3*a*). Such patterns indicate that biodiversity declines were not caused simply by loss of vulnerable taxa with notionally narrow thermal tolerances but were also owing to the rise of a small number of groups that were either tolerant to, or advantaged by, elevated soil temperatures. Oribatid mites, which increased in abundance by 56% in soil and 35% in leaf litter, have been found to be adapted to a wide range of temperatures [53] and appear to have higher heat tolerance than other key members of the soil and leaf litter fauna including Collembola and Protura [54], possibly owing to their hard exoskeletons giving them greater resistance to warming-induced desiccation [39]. A recent study of ant communities at our study site revealed that taxon-specific abundance responses to warming are at least partially controlled by variability in heat and desiccation tolerance among taxa [51]. Our results support this conclusion and show that similar mechanisms likely operate throughout entire communities of tropical soil and leaf litter fauna.

**Figure 4.**
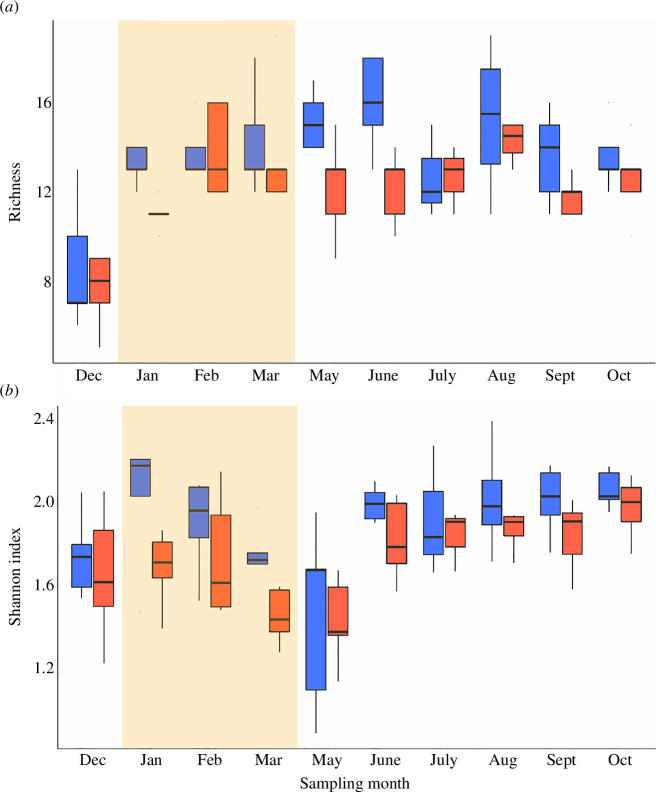
Soil fauna order level diversity and richness over time. (*a*) Mean total richness by month. (*b*) Mean total Shannon diversity by month. Box plots are standard Tukey plots, where the centre line represents the median, the lower and upper hinges represent the first and third quartiles and whiskers represent +1.5 the interquartile range. Blue box plots represent control plots, while red box plots represent warmed plots. The shaded areas represent the dry season (1 January to 1 April) where precipitation was < 50 mm per month.

In our study, the responses of soil fauna communities to experimental warming were strongly modulated by soil moisture across the seasonal cycle. Seasonal tropical forests, such as those at our study site, are characterized by strong seasonal patterns in rainfall (electronic supplementary material, figure S6) and in leaf phenology (loss of leaves during the dry season), factors that evidently had large modulating effects on the response of soil fauna to experimental warming. Seasonality was the primary driver of this moisture interaction because experimental warming did not directly affect moisture either in soil [52] or leaf litter (electronic supplementary material, figure S7). Soil fauna diversity declined with soil moisture levels from January through March, regardless of warming treatment (figure 4; electronic supplementary material, figure S5b), but the effect of warming on soil fauna diversity was significantly stronger during the dry season (January through March; effect size = 13.7%) than in the wet season (May to December; effect size = 6.1%). There was also seasonal variation in the positive effect of warming on total soil faunal abundance, with abundances in warmed plots 21.4% and 13.4% higher during the dry and wet seasons, respectively (figure 2*b*). This result contrasts with findings from a temperate forest where moisture and the interaction of moisture and temperature did not affect arthropod abundance [42]. A peatland warming study also found that warming-induced drying negatively impacted soil faunal communities [43]. When warming and drying were independently induced at higher latitudes, drought was the stronger predictor of changes in Collembola communities than warming, with warming having no significant effects [47,48]. This contrasts with our consistent finding of significant effects of warming on tropical soil fauna (including Collembola). A global meta-analysis reported that decreased moisture results in decreased soil faunal abundance (although tropical systems were notably under-represented; [25]). However, the increase in total faunal abundance we observed in the dry season matches observations made in other tropical forests, where the abundance of termites (part of order Blattodea) increased under prolonged drought [31]. We explain the enhanced effect of warming on total faunal abundance during the dry season by the increased abundance of leaf litter [55], an important habitat and substrate supply to soil fauna [56]. In seasonal tropical forests, including our own study site, leaf litterfall and litter standing crop peak during the dry season (electronic supplementary material, figure S7) [55]. Subsequently, during the early wet season, the accumulated litter decomposes rapidly and contributes to increased soil respiration [57]. Indeed, our observed peak in soil faunal abundance during the dry–wet season transition (figure 2*b*) followed the same trend observed for soil respiration at our experimental site [52] and in nearby forest [58]. Thus, the enhanced effect of warming on faunal abundance during periods of high leaf litter input points to an acceleration of soil process rates under warming, particularly when substrate availability is high [55]. Meanwhile, the diversity decline under drier conditions observed in this study indicates that stress associated with moisture limitation could increase the sensitivity of some tropical soil fauna to warming, and raises the possibility that the combined effects of warming and drought predicted with climate change [59] will have major consequences for soil biodiversity in the tropics.

Our results highlight the potential for losses of biodiversity from tropical soil and leaf litter environments under a warming climate, with likely ramifications for ecosystem function [26,28,29]. For example, Collembola and Isopoda both declined with warming in both soil and leaf litter at our study site, while Oribatida, one of the most abundant taxa, had a strong positive response to warming (electronic supplementary material, table 1; electronic supplementary material, table 2). These groups are important taxa within soil food webs, with complex detritivorous diets [60] that are flexible and responsive to substrate availability and community structure [29,61]. Several studies have found that experimental warming can increase rates of fungal grazing by Collembola [50], Isopoda [49] and Acari [39], indicating a tendency for warming to increase the degree of ‘top-down’ control over soil food webs via selective grazing [49,62]. This grazing does not necessarily decrease overall fungal biomass but puts selective pressure on fungal communities that favours certain fungal taxa. These ‘top-down’ effects on microbial communities have consequences for various soil processes, including respiration [49,62], suggesting that these mechanisms buffer the strength of positive climate feedbacks associated with soil C release to the atmosphere. Previous studies have demonstrated relationships—both positive and negative—between the abundance and grazing activity of soil fauna and soil process rates, including CO_2_ emission [63]. Thus, the changes in abundance of these detritivorous groups that we observed in our study are likely to cascade across trophic levels, with implications for rates of microbial grazing and organic matter decomposition. Indeed, the decline in diversity but not biomass of microbes at our study site suggests that different patterns of selective grazing by soil fauna may be occurring under warming [64].

An additional top-down control on the soil microbial community and soil function may come from the increased activity and abundance of termites and ants. Termites increased in abundance in soil and litter of warmed plots (electronic supplementary material, table 1; electronic supplementary material, table 2), suggesting high thermal resilience (e.g. high CT_max_), which is consistent with observations across global latitude gradients suggesting increased termite activity with warming based on their contribution to decomposition [31]. Ant community composition also shifted with warming at our study site, with increased abundance of *Wasmannia auropuctata*, a species known to predate other invertebrates [51]. Thus, given the widespread evidence from elsewhere that soil fauna affect soil functions [39,49,50], the large changes we observed in the composition and abundance of soil fauna under warming may represent top-down drivers of other soil changes: shifting the microbial community composition and reducing its diversity [64] and increasing soil CO_2_ emission [52]. Further study, especially at finer taxonomic resolution, is needed to elucidate the functional implications of these changes in tropical arthropods to warming.

Our results demonstrate a large effect of soil warming on arthropod community composition, diversity and abundance in a tropical forest, but require consideration in the context of our experimental design. By imposing plot-scale heating treatments, it is likely that changes in abundance that we detected resulted from both mortality and migration away from the warming plots. Thus, migratory responses to warming may be contributing to the effects observed in our study. For example, Poduromorpha appeared especially sensitive to warming, decreasing in abundance by 255% in soil. Most Poduromorpha species are euedaphic (living deeper in the soil, where temperatures are typically more stable) and their primary response to warming is to migrate even deeper. Indeed, vertical migration induced by short-term climate fluctuations (e.g. heat and drought) has been documented for an array of soil fauna [24,65–67]. However, we heated the entire soil profile to the bedrock, so deeper soil would not provide a cooler refuge in our experiment. Moreover, long-term climate warming will heat the entire soil profile [13], so that vertical migration to deeper, cooler soil is not a viable strategy. Our finding of a high sensitivity of Poduromorpha to elevated soil temperatures is consistent with results from a grassland warming study showing that euedaphic Collembola were highly sensitive to warming [45]. In contrast, Entomobryomorpha, which are mostly epigeic (surface-dwelling), increased with warming in our experiment, confirming predictions that this would be the group of Collembola least sensitive to warming. Migration itself is indicative of a stress response and a preference for lower temperatures that is thereby likely to translate into mortality as the entire landscape warms [68].

A limitation of our study design was the order-level identification of arthropod taxa, which limits our ability to infer finer-scale functional implications of our results given that some orders (e.g. Coleoptera) contain many species that vary in diet, metabolism and ecological roles. We prioritized a high number of observations over a high level of taxonomic resolution. This choice was driven by the difficulty of differentiating many tropical soil fauna groups at the genus and species levels [12] and justified by prior research indicating that diversity trends at higher taxonomic levels tend to parallel species-level trends [69–72]. Order-level data still provide substantial insight into arthropod assemblage responses to warming, even if functional implications at the species level remain elusive. Furthermore, our data can give some insight into species-level effects if they were quantified across the entire arthropod assemblage (rather than within orders). For example, in the case of richness and diversity, we believe it to be parsimonious that species-level effects would be qualitatively consistent with order-level effects: a substantial *increase* in species-level diversity within orders under warming would be needed to offset the marked declines in order-level diversity that we observed. Such increases are unlikely, given the numerous reports of species-level diversity declines under warming in other ecosystems [1,3,5,16], meaning that the latent species-level responses at our study site should be similar to (or greater than) order-level responses. In the case of community composition, species-level composition must have been unaffected by warming, given that order-level composition was affected and that a given species always belongs to the same order. Even so, we recognize the value of characterizing species-level responses in future work. This task has been historically challenging for tropical soil fauna but will become increasingly feasible in the future as DNA barcode libraries are expanded and costs of metabarcoding decrease.

The declines in order-level invertebrate diversity and richness, and the community shift that were observed with warming in our study (figures 1–3) provide evidence that supports the prediction that tropical arthropod communities may be more sensitive to warming than their temperate counterparts [11,14]. At the same time, warming increased total invertebrate abundance as specific taxa became dominant (figures 2 and 3), contrasting with findings from warming experiments performed at higher latitudes that found no effect on diversity [37–40] and decreased abundance [39,41,42]. Our results go further by demonstrating that diversity declines under warming are exacerbated by seasonal drying (figure 4; electronic supplementary material, figure S5), with large implications for the impacts of warming and drought on tropical soil function. In summary, we provide experimental evidence that supports observed correlations between climate warming and arthropod diversity decline in the tropics [5,6,16]. However, unlike observational studies, which show declines as they occur, experimental studies predict a future that can still be averted: biodiversity still abounds at sites like ours, and we still have a chance to protect it [73] and the valuable services it provides.

## 4. Methods

### (a) Study site

The SWELTR experiment (Soil Warming Experiment in Lowland TRopical forest) is located in a mature secondary (>100 years) semi-deciduous lowland tropical forest on BCI in Central Panama [52]. The island has been well protected from land use change for the past 100 years, and the surrounding mainland is similarly depopulated and protected lowland tropical forest. The mean annual temperature is 26°C (with low seasonal variation of *ca* 1°C) and the mean annual rainfall is 2600 mm, with a strong dry season from January through March [74]. The site is situated on a moderately weathered Inceptisol, a soil type that covers 14% of total land area in the tropics (Ultisols and Oxisols account for 20% and 23%, respectively; [75]). The dominant canopy-forming trees and main source of leaf litter on our plots are *Anacardium excelsum* and *Poulsenia armata*.

The experiment consists of five pairs of 25 m^2^ circular plots situated in a 1 ha area, with one plot per pair warmed to 1.2 m depth by 4°C above the temperature of the adjacent control plot, based on the average temperature across the soil profile (0–1.2 m depth). Warming began on 1 November 2016 [52], 1139 days prior to first soil fauna collection. The precipitation over the course of the year when this study was conducted was nearly at the median and mean for the area since recording began in 1925 [76,77]. All measurements were performed within the plot area where the soil is evenly heated throughout the year (annual mean surface warming by 3°C, annual mean whole-profile warming by 4°C; [52]). We found no effect of warming on annual soil moisture at our experimental site [52]. The warming experiment design that we deployed follows successfully implemented experiments elsewhere, which also demonstrated evenly heated soil within plot areas of the same size [78,79].

### (b) Invertebrate sampling and extraction

We collected soil and litter samples from the experimental plots on 10 dates from 14 December 2019 to 1 October 2020. On each sampling occasion we selected three subsampling locations per plot, with each of these positioned *ca* 2 m from the plot centre and not directly over the heating cables. Soil subsamples consisted of 10 cm × 10 cm-wide and 5 cm-deep portions of the surface soil, removed with a serrated knife and small trowel. Both soil and litter subsamples were homogenized in the field to yield one soil and one litter sample per plot. New sets of subsample locations within each plot were selected on each sampling date, and total sampled area per month represented approximately 0.15% of the total warmed area per plot, so sampling did not affect existing soil communities. Owing to logistical constraints imposed by the COVID-19 pandemic, on 1 July 2020 we could only sample three of the five plot pairs, and four of the five pairs on 3 August 2020.

The samples were taken from the field, weighed and placed in Tullgren funnels within 4 h of sampling. The mesh in the litter extraction funnels had an aperture of 9 mm × 9 mm while the mesh in the soil extraction funnels had an aperture of 2 mm × 2 mm. Invertebrates were extracted for 72 h under 25 W incandescent bulbs into 70% ethanol and thereafter refrigerated. Soil and litter were returned to the respective experimental plots after invertebrate extraction. Tullgren funnels used in isolation from other techniques are not ideal for some taxa [80], although they are appropriate for soil arthropods in this experimental comparison context [34,38,39,56].

We identified invertebrates to Order level, although in some cases higher taxonomic levels were used. Identifications of the subclass Acari were split into Oribatida and a group consisting of all other mites. Chilopoda, Diplopoda and Clitellata (Annelida) had relatively low abundances and were not identified beyond Class. Diversity at higher taxonomic levels reflects species-level changes [69,70], and we expect that differences in diversity and community composition at the order level are likely to reflect differences in functional diversity. We converted soil invertebrate abundance to count per 1000 g dry soil (using gravimetric soil moisture and sample mass). Owing to constraints imposed by the COVID-19 pandemic, litter moisture values could not be measured, so count data could not be converted to count per dry mass of litter; however, this does not affect our estimates of ordinal taxonomic richness or Shannon diversity based on relative abundances. Warming did not affect leaf litter moisture on the SWELTR plots during our study period. Unidentified specimens (largely insect larvae and unsegmented worms)—a group that constituted 0.38% (s.e. ± 0.07) of soil and 0.17% (s.e. ± 0.024) of litter samples, respectively—were excluded from all analyses except for those that involved total invertebrate abundance. The invertebrates collected during this study are stored by taxon, plot number and collection date and held in the Smithsonian Tropical Research Institute collection.

### (c) Soil and leaf litter climate data

We determined soil and leaf litter temperature and moisture for all sampling locations. Leaf litter temperature was determined using a thermometer probe (HI98509; Hanna Instruments, USA) for the leaf litter within each 20 cm × 30 cm sampling area prior to its collection. Leaf litter moisture was determined for the litter-standing crop from each experimental plot on two occasions, during the wet season (November 2022) and dry season (April 2023). For moisture determination, the litter standing crop was collected from two 50 cm × 50 cm (0.25 m^2^) quadrants per plot (*n* = 10 plots) and dried at 60°C for 48 h. Therefore, we report both leaf litter standing crop and leaf litter moisture for all sites during the dry and wet seasons (electronic supplementary material, figure S7). Soil temperature and volumetric moisture were determined at each sampling date, using a moisture probe (Thetaprobe; Delta-T Devices, UK) and temperature probe (HI98509; Hanna Instruments, USA).

### (d) Statistical analyses

We defined the dry season as months with <50 mm rainfall (January through March), with the remaining wet season months during our sampling period having >140 mm rainfall (we did not sample in April, which was a transitional month; [76,77]). We calculated Shannon, Simpson and Pielou’s Evenness indexes using the ‘Vegan’ package [81] in R v. 4.1.1 [82]. All metrics showed similar results (see electronic supplementary material, table 3), so we focused on the Shannon index as a measure of invertebrate diversity.

Treatment (warming) and seasonal (soil moisture) effects on Shannon diversity were tested using a linear mixed effects model with warming treatment, soil moisture and the warming–soil moisture interaction as fixed effects, and plot pair as a random intercept effect. However, this model was overfitted, with no variation attributed to plot pair; thus, we simplified the model by removing the random effect. We used ANOVA to determine the significance of each of the predictor variables (fixed effect terms). Treatment and seasonal effects on ordinal taxonomic richness and total abundance were analysed in the same way (including the initial tests of mixed effects models), but with generalized linear models and Poisson distributions. We did not use a repeated-measures analysis because each sub-sample collection location was independent. To confirm significant results, we reran analyses excluding mites (electronic supplementary material, figure S2), which were the most abundant taxa, and again excluding 14 orders that only occurred in <10% of soil samples. There is the potential for the effects of warming on soil and leaf litter fauna to result from larger, more mobile fauna migrating into or out of the warmed plots, based on their thermal tolerance, rather than increased mortality within the plot. However, this still represents a true effect of warming on those fauna; we expect that low thermal tolerance (i.e. migrating out of the plot) would result in mortality over time if there weren't a nearby ambient temperature plot to which they could migrate.

To visualize the effect of warming and moisture on invertebrate community composition, we used non-metric multidimensional scaling (NMDS) ordination based on relative abundances and Bray–Curtis dissimilarity matrices (Vegan package [81]; R [82]). Auto-transformation was disabled, maximum number of iterations was set to 1000 and the analysis was limited to two dimensions. We then carried out a PERMANOVA to complement the NMDS. Bootstrapping was accomplished with a PERMANOVA test (adonis function) and the assumption for the homogeneity of multivariate dispersion was tested with an ANOVA of distances (vegdist function), Vegan package [81], R [82]. The assumption for the homogeneity of multivariate dispersion was met, with no significant differences between variation within treatments (*p* = 0.1724). To test whether the transformation influenced our results, we ran NMDS with three different transformations (‘log’, ‘Hellinger’ and ‘normalize’) on both relative and absolute abundance data. The NMDS models based on all combinations of transformed data gave the same results as the model based on untransformed relative abundance, but exhibited greater stress, thus they were not selected for the final analysis. All analyses were performed in R v. 4.1.1 [82].

### (e) Estimating species-level effects

We estimated the potential consequences of warming-induced declines in the richness of arthropod orders for the richness of arthropod species at our site by drawing on, and extrapolating from, the comprehensive species-level inventory of arthropods at the San Lorenzo Protected Area (SLPA) [12], which is situated *ca* 20 km northwest of our study site on BCI. In doing so, we first calculated species richness : abundance (i.e. counts) ratios for each order from the observed 0.48 ha plot-level species richness and abundance data from the SLPA inventory study.

We then multiplied the total abundance of each order (summed across replicate plots and over all time points) in our own control samples by the most appropriate species richness : abundance ratio available in the SLPA inventory to give an estimate of species richness for each order in our samples. For taxa for which there was no obviously appropriate species richness : abundance ratio in the SLPA data (e.g. low abundance soil fauna such as Palpigradi, Pseudoscorpiones and Symphyla), we used the overall arthropod species richness : abundance ratio. Through this procedure, we arrived at estimates of 261 species in soil extracts and 337 species in litter extracts. Intra-order species richness values ranged from 1 species (numerous Orders) to 79 species (Hymenoptera) for soil and from 1 species (numerous Orders) to 85 species (‘Acari other’) for litter. Next, we identified several orders as particularly vulnerable to major decline and potentially local extinction under warming; these orders were those showing statistically significant (*p* < 0.05) declines in abundance with effect sizes of >50%. For soil, there were 10 such orders, while for litter there were 11 such orders (plus the ‘unknown’ taxon). Following this, we calculated the decline in total species richness of the soil and litter arthropod assemblages, as sampled, if one or two of the notionally vulnerable orders were excluded.

## Data Availability

Data files are hosted by Dryad [83]. Supplementary material is available online [84].
